# Effect of *Lactobacillus rhamnosus* GG on Energy Metabolism, Leptin Resistance, and Gut Microbiota in Mice with Diet-Induced Obesity

**DOI:** 10.3390/nu12092557

**Published:** 2020-08-24

**Authors:** Yu-Chieh Cheng, Je-Ruei Liu

**Affiliations:** 1 Department of Animal Science and Technology, National Taiwan University, Taipei 106, Taiwan; r03626014@ntu.edu.tw; 2 Institute of Biotechnology, National Taiwan University, Taipei 106, Taiwan; 3 Center for Biotechnology, National Taiwan University, Taipei 106, Taiwan; 4 Agricultural Biotechnology Research Center, Academia Sinica, Taipei 106, Taiwan

**Keywords:** *Lactobacillus rhamnosus*, gut microbiota, leptin resistance, obesity

## Abstract

Obesity is closely associated with various metabolic disorders, including leptin resistance, which is characterized by high circulating leptin levels. Probiotics can decrease circulating leptin levels by alteration of the gut microbiota. Thus, they may have anti-obesogenic effects. In this study, the effects of administration of a probiotic bacterium, *Lactobacillus rhamnosus* GG (LGG), on gut microbiota and modulation of leptin resistance were evaluated in mice. Male Balb/C mice aged 7 weeks were fed either a normal diet (ND), high-fat diet (HFD), HFD supplemented with low-dose LGG (10^8^ CFU/mouse/day), or HFD supplemented with high-dose LGG (10^10^ CFU/mouse/day) for 10 weeks. Significantly increased body weight, epididymal fat weight, and decreased leptin responsiveness to exogenous leptin treatment and ratio of villus height to crypt depth were observed in the HFD-fed mice compared to the ND-fed mice. Moreover, a remarkable increase in the proportion of Proteobacteria and ratio of Firmicutes/Bacteroidetes in the fecal microbiota were also observed in the HFD-fed mice. Supplementation of HFD with high-dose LGG restored exogenous leptin responsiveness, increased the ratio of villus height to crypt depth, and decreased the proportion of Proteobacteria in fecal microbiota. These findings suggest that LGG supplementation might alleviate leptin resistance caused by an HFD through the improvement of the digestive health of the host.

## 1. Introduction

Obesity is defined by the World Health Organization (WHO) as a body mass index (BMI) of 30 or more. It is a chronic disease that has reached epidemic proportions globally. According to the data released by the WHO, in 2016, more than 1.9 billion adults (18 years and older) were overweight, and, of these, over 650 million were obese [1]. Obesity is closely associated with various metabolic disorders, including hypertension, type 2 diabetes mellitus, cardiovascular disease, and nonalcoholic fatty liver, which are highly associated with mortality. The fundamental cause of obesity is an imbalance between energy intake and energy expenditure. Energy homeostasis is controlled by the hypothalamus in the central nervous system, which receives signals from the adipose tissue, digestive system, and endocrine system and manages appetite via the nervous system [2].

Leptin, one of the appetite-regulating hormones produced by adipose tissue, facilitates control of food intake and energy expenditure through binding to leptin receptors in the hypothalamus, leading to the suppression of fat accumulation and a reduction in body weight [3]. Circulating leptin levels are related to body fat mass. However, high circulating leptin levels are associated with leptin resistance. Previous studies found that obese humans and mice had severe hyperleptinemia that was recognized as leptin resistance. The development of leptin resistance in the central nervous system may contribute to this metabolic disorder. Although the reasons for leptin resistance are not well known, some mechanisms have been proposed. The upregulation of cytokine signaling-3 (*SOCS3*) and protein-tyrosine phosphatase-1B (*PTP1B*) gene expression is associated with the development of leptin resistance. Both SOCS-3 and PTP1B are possible mediators that contribute to reduced long-form leptin receptor LepRb signaling, which reduced leptin sensitivity by inhibiting the phosphorylation of signal transducer and activator of transcription-3 (STAT3), an intracellular leptin-signaling mediator [4,5].

In 2004, Backhed et al. [6] showed that the gut microbiota is a crucial environmental factor that affects energy homeostasis and fat accumulation. After this, numerous studies have proven the association between the gut microbiota and energy metabolism [7,8,9]. Moreover, the effect of the gut microbiota on circulating leptin levels and energy balance by regulation of peptides in the hypothalamus, including SOCS3, has been reported in the literature [4]. Modulation of the gut microbiota by probiotics intervention may enhance host health [10]. Some studies indicate that the administration of probiotics decreases circulating leptin levels by alteration of the gut microbiota and thus suggest that probiotics may have anti-obesogenic effects [11,12,13].

Interestingly, not only the viable probiotic cells but also the non-viable probiotic metabolites may have anti-obesogenic effects. Sousa et al. [14] demonstrated that injection of a *Lactobacillus* supernatant into the cerebral ventricles of rats led to a decrease in body weight and an increase in leptin expression in the central neurons and adipose tissue. Therefore, the study brought to light that probiotics have an association with leptin resistance mechanisms due to their influence on leptin expression in central neurons.

*Lactobacillus rhamnosus* GG (LGG) is one of the most widely studied probiotic bacterial strains, especially in the area of metabolic syndrome. The anti-obesogenic and antidiabetic effects of LGG can be attributed to its improvement of insulin-resistance by modulating the gut microbiota in instances of diet-induced obesity in mice [12,15]. Recently, Darby et al. [16] reported that the administration of LGG induced an elevated level of NADPH oxidase (Nox1), which in turn activated the nuclear factor erythroid 2-related factor 2 (Nrf2) cell signaling pathway, resulting in the upregulation of leptin expression. However, there is still a lack of data on the effects of LGG administration on the modulation of leptin resistance.

The purpose of this study was to expand the comprehension of the relationships among the LGG, leptin resistance, and host gut microbiota in diet-induced obesity in mice.

## 2. Materials and Methods

### 2.1. Preparation of LGG

LGG (ATCC 53013) was obtained from the American Type Culture Collection (ATCC; Manassas, VA, USA) and was cultured in de Mann–Rogosa–Sharpe (MRS) broth (Difco, Detroit, MI, USA) at 37 °C without shaking. After 24 h of incubation, the LGG cultures were centrifuged at 6000× *g* for 10 min at 4 °C, and the cell pellets were washed twice with sterile phosphate-buffered saline (PBS; 0.1 M, pH 7.0). Then, the LGG cells were re-suspended in PBS (0.1 M, pH 7.0) to yield a final bacterial concentration of 10^8^ or 10^10^ CFU/100 μL. It was prepared immediately before its administration to each mouse by gavage.

### 2.2. Animal Care and LGG Administration

Male Balb/C mice were purchased from BioLASCO (Taipei, Taiwan) at 7 weeks of age and allowed to acclimatize for one week before the start of the experiment. They were kept on a 12-h light/dark cycle in the Institute of Biotechnology Animal Room at National Taiwan University. The animal room was maintained at 25–29 °C and relative humidity between 50 and 70%. After one week of acclimatization, mice were randomly divided into four groups, with 10 animals in each group.

The first group ate a normal diet consisting of standard rodent chow (Laboratory Rodent Diet 5001; 3.10 kcal/g with 29% energy derived from protein, 13% from fat, and 56% from carbohydrate; PMI Nutrition International, St. Louis, MO, USA) and served as the normal diet (ND) control group. The second group ate a high-fat diet and served as the high-fat diet (HFD) control group. The high-fat diet consisted of TestDiet 58Y1 (5.10 kcal/g with 18.1% energy derived from protein, 61.6% from fat, and 20.3% from carbohydrate; PMI Nutrition International, St Louis, MO, USA). The other two groups served as the probiotic groups and received either a high-fat diet supplemented with low-dose LGG (HFD+LGG-LD; 10^8^ CFU/mouse/day) or a high-fat diet supplemented with high-dose LGG (HFD+LGG-HD; 10^10^ CFU/mouse/day). The LGG-treated mice were administered LGG daily by oral gavage at a dose of 10^8^ or 10^10^ CFU/mouse/day of LGG, whereas mice in the control groups received sterile PBS as the vehicle by daily oral gavage. The experiment ran for 10 weeks. During the experiment, the diets and water were provided ad libitum, and body weight and feed intake were measured weekly. The animal studies and protocols were approved by the Institutional Animal Care and Use Committee of National Taiwan University (NTU104-EL-00055).

### 2.3. Leptin Resistance Test

The leptin resistance test was performed according to the procedure proposed by Schele et al. [4]. Recombinant mouse leptin (R&D System, Inc., Minneapolis, MN, USA) was dissolved in 0.9% saline and sterile filtered through a 0.22-μm filter. Three days before the mice were humanely killed, the mice in each group were divided into two subgroups and intraperitoneally administered leptin (1 µg/g body weight) or vehicle twice daily for 3 days. Body weights and feed intake were measured twice daily before and after the leptin injection. The mice were killed the day after the final leptin injection. Blood samples were collected by cardiac puncture, and serum leptin concentrations were measured using the Mouse Leptin DuoSet ELISA Kit (R&D System, Inc., Minneapolis, MN, USA).

### 2.4. Quantitative Real-Time Polymerase Chain Reaction (qRT-PCR) Analysis of SOCS3 Expression

Forty-five minutes after the last intraperitoneal injection of leptin, the hypothalami were collected, dissected, and immediately frozen in liquid nitrogen. Total RNA was extracted using the GENEzolTM TriRNA Pure Kit (Geneaid Biotech Ltd., New Taipei, Taiwan), and the complementary DNA (cDNA) was synthesized by reverse-transcribing 0.2 μg of RNA using the QuantiTect Reverse Transcription kit (Qiagen Inc., Valencia, CA, USA). The mRNA expression levels of *SOCS3* were measured by qRT-PCR using gene-specific primers (forward primer: 5′-ATGGTCACCCACAGCAAGTTT-3′; reverse primer: 5′-TCCAGTAGAATCCGCTCTCCT-3′) with the Luminaris Color Higreen High ROX qPCR Master Mix kit (Thermo Fisher Scientific, Inc., Waltham, MA, USA) and normalization to the housekeeping gene β-actin.

### 2.5. Histological Observation of Liver and Intestine

Liver and ileum samples were collected, fixed in 10% neutral buffered formalin (NBF) for 24 h, embedded in paraffin, and processed to section. Both liver and ileum sections were stained with hematoxylin and eosin (H&E) to verify the histopathologic diagnosis. Villi length and crypt depth in the ileum were measured using ImageJ software. Sudan black B (SBB) staining was performed for the observation of lipid droplet distribution in mice liver tissue. For the SBB staining, the NBF-fixed liver tissues were processed for cryosectioning and SBB staining according to the method described by Georgakopoulou et al. [17].

### 2.6. Metagenomic Analysis of Fecal Microbiome

Fecal samples were collected at the end of the experiment. The extraction of fecal bacterial genomic DNA was performed using the QIAamp Fast DNA Stool Mini tool kit (Qiagen Inc., Valencia, CA, USA). The standard 16S rRNA gene primers, 341F (5′-CCTACGGGNGGCWGCAG-3′) and 805R (5′-GACTACHVGGGTATCTAATCC-3′), which can be used to amplify the nucleotide sequence from the hypervariable regions V3 to V4 of the 16S rRNA gene of a wide variety of bacterial taxa, was used for polymerase chain reaction (PCR) to amplify the partial 16S rRNA gene sequence of the fecal bacteria [18]. The resultant PCR products were excised from the gel and purified using the QIAquick Gel Extraction Kit (Qiagen Inc., Valencia, CA, USA). DNA libraries were prepared for sequencing using the TruSeq DNA Sample Preparation Kit (Illumina, San Diego, CA, USA). Library DNA concentration was measured using the QuantIT kit (Molecular Probes, Carlsbad, CA, USA) and paired-end sequenced (2 × 300 bp reads) on the Illumina MiSeq platform (Illumina, Inc., San Diego, CA, USA) as a service provided by Genomics BioSci & Tech. Co., Ltd. (Taipei, Taiwan). The readouts obtained from the sequencing were analyzed by using the MacQIIME (1.8.0) pipeline [19]. Briefly, the paired reads were merged into amplicon sequences. Then, these amplicon sequences were dereplicated and clustered into operational taxonomic units (OTUs) at 99% sequence similarity and taxonomically assigned using the SILVA SSU database version 119.1 (The SILVA ribosomal RNA database project, Bremen, Germany). Post analysis including alpha, beta diversity, and taxonomic assignment of microbiota was conducted using the MacQIIME [19].

### 2.7. Statistical Analysis

The data were analyzed using SPSS version 25 software (IBM SPSS, New York, NY, USA). One-way analysis of variance (one-way ANOVA) followed by Tukey’s test was used to detect the differences among the means of the different treatment groups. Student’s t-test was used to detect the differences between the treatment and control groups. A *P*-value of less than 0.05 was considered significant. All results were expressed as means ± standard deviation.

## 3. Results

### 3.1. Effect of Administration of LGG on Body Weight and Feed Intake in Diet-Induced Obesity in Mice

**Figure 1:**
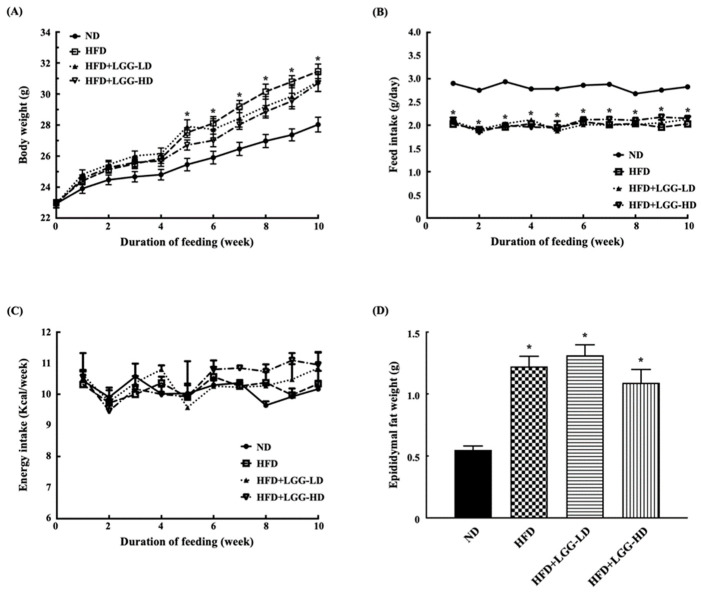
Effect of administration of *Lactobacillus rhamnosus* GG (LGG) on physiologic parameters in mice with diet-induced obesity. (**A**) Body weight. (**B**) Feed intake. (**C**) Energy intake. (**D**) Epididymal fat level. ND: normal diet; HFD: high-fat diet; HFD+LGG-LD: high-fat diet supplemented with low-dose LGG (10^8^ CFU/mouse/day); HFD+LGG-HD: high-fat diet supplemented with high-dose LGG (10^10^ CFU/mouse/day). All data are expressed as mean ± SD (*n* = 10). Bar marked with a star * means that it is significantly different from the ND group at *p* < 0.05.

During the first 4 weeks of the experiment, the body weights of the mice in each group increased steadily, and there was no significant difference among them. From the fifth week of the experiment, the body weights of the three groups of mice fed a high-fat diet were significantly higher than those of the mice fed a normal diet. However, there was no significant difference in the body weight among the three groups fed a high-fat diet (Figure 1A). To examine the changes in feeding behavior, we measured feed intake and energy intake. The feed intake in the three groups of mice fed a high-fat diet was significantly lower than that of the mice fed a normal diet (Figure 1B). However, there was no significant difference in the energy intake among the four groups over the 10 weeks of the experiment (Figure 1C). Additionally, the epididymal fat weights of the three groups of mice fed a high-fat diet were significantly higher than those of the mice fed a normal diet (Figure 1D). However, there was no significant difference in the epididymal fat weight among the three groups fed a high-fat diet (Figure 1D).

**Figure 2:**
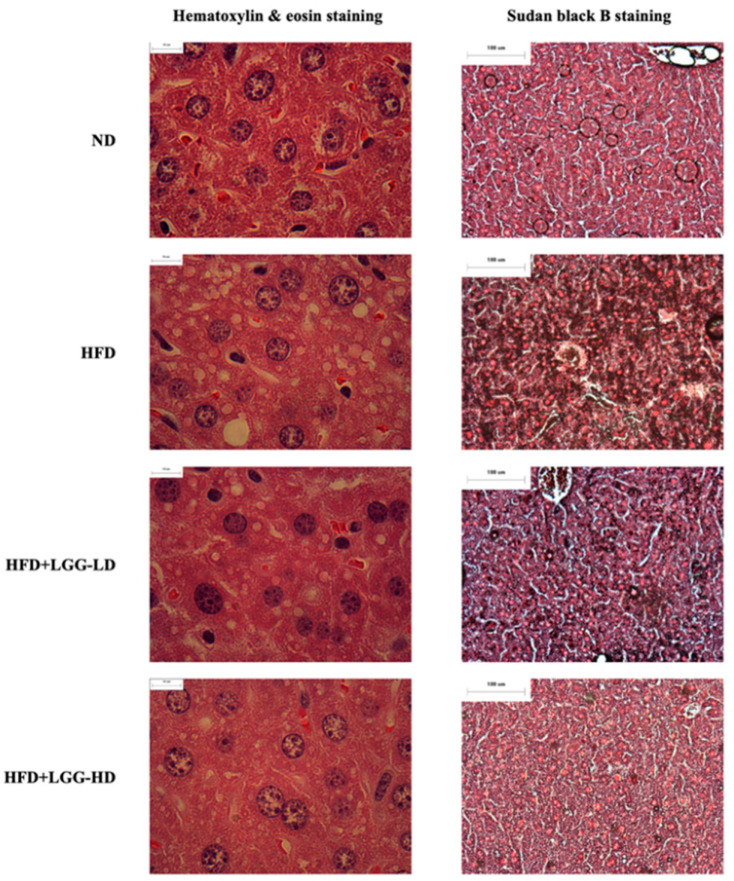
Effect of administration of *Lactobacillus rhamnosus* GG (LGG) on hepatic morphology and lipid accumulation in mice with diet-induced obesity mice. ND: normal diet; HFD: high-fat diet; HFD+LGG-LD: high-fat diet supplemented with low-dose LGG (10^8^ CFU/mouse/day); HFD+LGG-HD: high-fat diet supplemented with high-dose LGG (10^10^ CFU/mouse/day).

H&E staining and SBB staining were performed to confirm the effect of a high-fat diet supplemented with LGG on the hepatic morphology and lipid accumulation in mice. The liver specimens from mice fed a high-fat diet had more lipid accumulation than those from mice fed a normal diet or a high-fat diet supplemented with LGG. Moreover, mice fed a high-fat diet exhibited microvesicular and macrovesicular liver steatosis, whereas the hepatic histology of mice fed the HFD+LGG-HD diet showed fewer hepatic lipid droplets (Figure 2).

### 3.2. Effect of Administration of LGG on the Serum Leptin Concentrations, Leptin Resistance, and Hypothalamic SOCS3 Expression Levels in Mice

**Figure 3:**
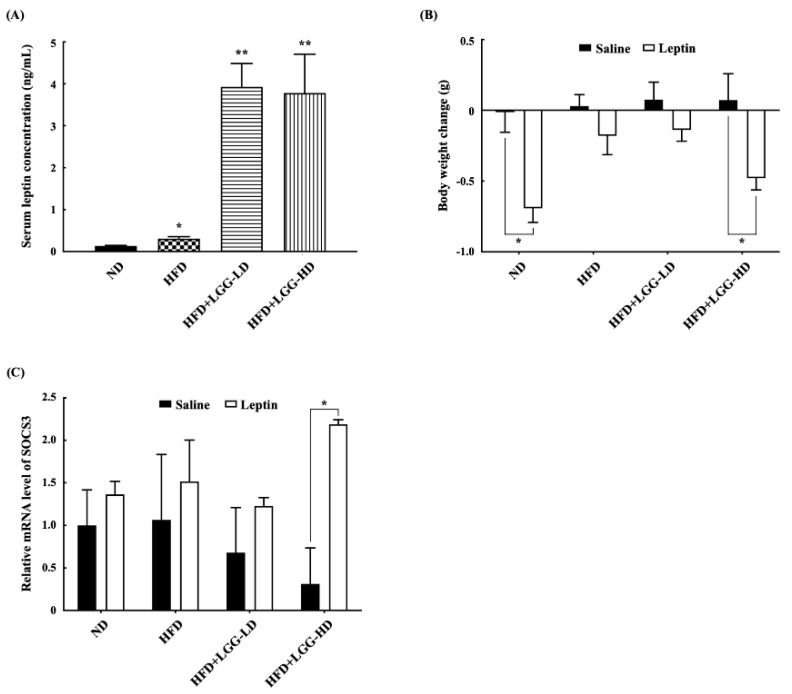
Effect of administration of *Lactobacillus rhamnosus* GG (LGG) on serum leptin concentrations and leptin responsiveness to exogenous leptin treatment of mice with diet-induced obesity. (**A**) Serum leptin concentrations. Bar marked with a star * or double stars ** means that it is significantly different from the ND group at *p* < 0.05 or *p* < 0.01, respectively. (**B**) Body weight change after treatment with exogenous leptin. Bar marked with a star * means that it is significantly different from the control (saline) at *p* < 0.05. (**C**) Relative hypothalamic mRNA expression level of *SOCS3* gene. The data are expressed as relative fold change compared with the ND group given saline, which is set as 1. Bar marked with a star * means that it is significantly different from the control (saline) at *p* < 0.05. ND: normal diet; HFD: high-fat diet; HFD+LGG-LD: high-fat diet supplemented with low-dose LGG (10^8^ CFU/mouse/day); HFD+LGG-HD: high-fat diet supplemented with high-dose LGG (10^10^ CFU/mouse/day). All data are expressed as mean ± SD (*n* = 5).

At the end of the experiment, the serum leptin levels and leptin sensitivity of the mice were determined. The serum leptin concentrations of the mice in the ND group were significantly lower than those of the mice in the HFD, HFD+LGG-LD, or HFD+LGG-HD groups (Figure 3A).

The effect of administration of LGG on leptin resistance was evaluated by using an acute method since leptin resistance prevents exogenous leptin treatment from promoting weight loss. The body weight change in mice after treatment with leptin intraperitoneally for 3 days was determined. During the 3 days of exogenous leptin treatment, the feed intake of the mice did not differ among the groups (results not shown). Exogenous leptin treatment did not cause significant weight loss in the mice fed a high-fat diet or those fed an HFD+LGG-LD diet. However, exogenous leptin treatment caused a marked and significant body weight reduction in the mice fed a normal diet and HFD+LGG-HD diet (Figure 3B). These results indicate that leptin responsiveness was reduced in mice fed a high-fat diet, and the administration of LGG at a high dose increased leptin sensitivity in the mice with diet-induced obesity.

The hypothalamic mRNA expression levels of the *SOCS3* gene were determined at the end of the experiment. In the subgroups given saline (vehicle), there was no significant difference in hypothalamic *SOCS3* expression among the various diet groups. Similarly, in the subgroups given exogenous leptin, there was also no significant difference in hypothalamic *SOCS3* expression in the normal diet, high-fat diet, and HFD+LGG-LD groups. However, the hypothalamic *SOCS3* expression in the HFD+LGG-HD group given exogenous leptin was significantly higher than that in the HFD+LGG-HD group given saline, indicating that the mice fed an HFD+LGG-HD diet were more sensitive to exogenous leptin (Figure 3C).

### 3.3. Effect of Administration of LGG on the Histological Morphology of the Ileum

**Figure 4:**
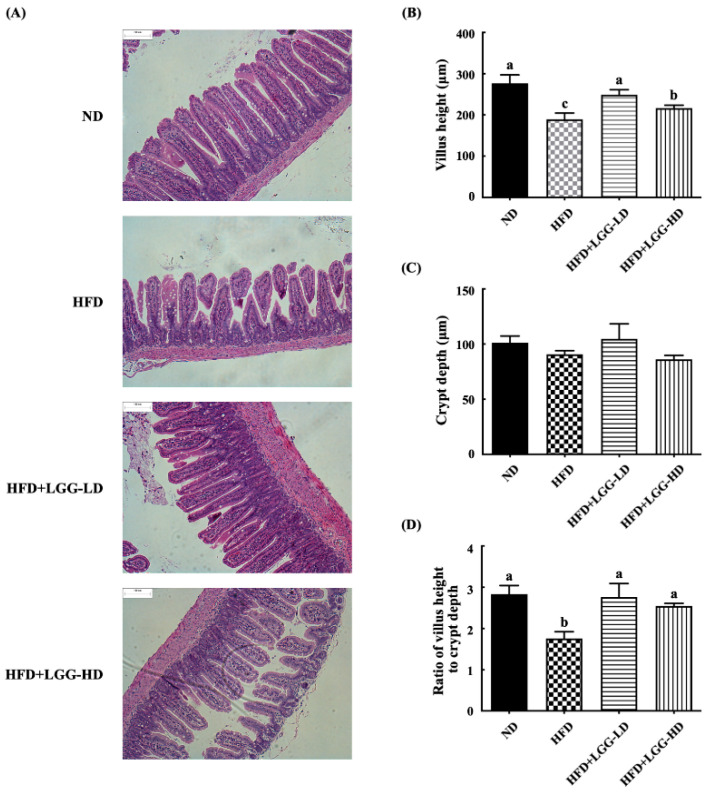
Effect of administration of *Lactobacillus rhamnosus* GG (LGG) on the histological morphology of the ileum in mice with diet-induced obesity. (**A**) Histological morphology of ileum. (**B**) Villus height. (**C**) Crypt depth. (**D**) Ratio of villus height to crypt depth. ND: normal diet; HFD: high-fat diet; HFD+LGG-LD: high-fat diet supplemented with low-dose LGG (10^8^ CFU/mouse/day); HFD+LGG-HD: high-fat diet supplemented with high-dose LGG (10^10^ CFU/mouse/day). ^a,b,c^, Bars marked with different letters are significantly different at *p* < 0.05.

Histological sections of H&E-stained ileum were examined to evaluate the effect of the high-fat diet and supplementation with LGG on the intestinal mucosa (Figure 4). The mice in the ND group showed normal histological morphology of the ileum, exhibiting a densely packed population of intact villi with a ratio of villus height to crypt depth of 2.83 ± 0.36. The mice in the HFD group showed decreased villus height and moderate villous blunting, with a ratio of villus height to crypt depth of 1.76 ± 0.29. Supplementation with LGG, whether at a low dose or high dose, significantly reduced the pathological alterations caused by the high-fat diet. Furthermore, the ratio of villus height to crypt depth was increased to normal levels (2.76 ± 0.57 and 2.54 ± 0.11, respectively).

### 3.4. Effect of Administration of LGG on Fecal Microbiota

**Figure 5:**
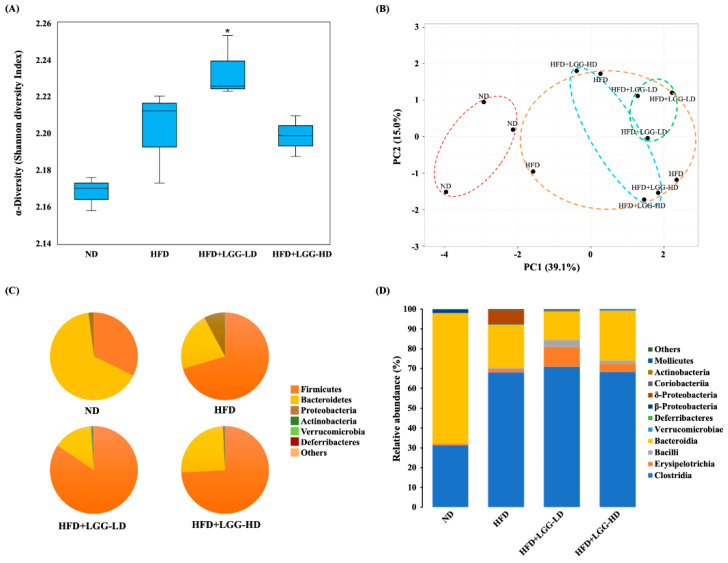
Effect of administration of *Lactobacillus rhamnosus* GG (LGG) on the fecal microbiota of mice with diet-induced obesity. (**A**) Boxplot representation of Shannon diversity index. Bar marked with a star * means that it is significantly different from the ND group at *p* < 0.05. (**B**) Two-dimensional plot of principal component analysis. (**C**) Fecal microbial composition at phylum level. (**D**) Fecal microbial composition at class level. ND: normal diet; HFD: high-fat diet; HFD+LGG-LD: high-fat diet supplemented with low-dose LGG (10^8^ CFU/mouse/day); HFD+LGG-HD: high-fat diet supplemented with high-dose LGG (10^10^ CFU/mouse/day).

The fecal microbiota compositions of the mice were determined by high-throughput sequencing of the 16S rRNA gene using the Miseq Illumina platform. The MiSeq sequencing yielded a total of 2.4 × 10^7^ reads, with an average size of 298.37 bp. The alpha diversity was estimated by the Shannon diversity index, whose highest values indicated greater bacterial diversity. Shannon diversity showed no significant differences between the ND and HFD groups, but the HFD+LGG-LD group showed an increase in Shannon diversity when compared to the ND group (Figure 5A). Figure 5B shows the principal component analysis (PCA) of the beta diversity associated with microbiota variance for mice fed a normal diet or a high-fat diet with or without LGG. The principal components represented an accumulated variance of 54.1% (PC1 39.1% and PC2 15.0%). According to the PC1 versus PC2 plot, the fecal microbiota compositions of mice fed a normal diet differed from those fed a high-fat diet. The fecal microbiota compositions of mice fed a normal diet were grouped on the left side of the chart along the PC1 axis. In contrast, the fecal microbiota compositions of the mice fed a high-fat diet with or without LGG supplementation showed a wider dispersion, indicating differences in beta diversity.

The composition and abundance of the fecal microbiota of different experimental groups are shown in Figure 5C,D. On the phylum level, Bacteroidetes and Firmicutes were the two dominant taxa in all groups (Figure 5C). In the mice fed a normal diet, the most numerous were Bacteroidetes (65.6%), followed by Firmicutes (32.3%). However, in the mice fed a high-fat diet, Firmicutes was more abundant (70.4%) than Bacteroidetes (21.7%). The ratios of Firmicutes/Bacteroidetes differed between the mice fed a normal diet (0.49) and those fed a high-fat diet (3.24). Moreover, in mice fed a high-fat diet, the proportion of the microbiota from the phylum Proteobacteria (7.4%) increased, which differed from that of the mice fed a normal diet (1.7%). Dietary supplementation with low-dose LGG and high-dose LGG in the high-fat diet decreased the microbial proportion from the phylum Proteobacteria to 0.08% and 0.10%, respectively. In addition, supplementation with low-dose LGG in the high-fat diet decreased the proportion of Bacteroidetes to 14.3% and increased the proportion of Firmicutes to 84.3%. In contrast, supplementation with high-dose LGG increased the proportion of Bacteroidetes to 25.0% and increased the proportion of Firmicutes to 74.1%. The ratios of Firmicutes/Bacteroidetes in the HFD+LGG-LD group and the HFD+LGG-HD group were 5.90 and 2.96, respectively.

On the class level, we found that the fecal microbiota compositions of the mice fed a normal diet and those fed a high-fat diet also differed (Figure 5D). The top three classes in order of abundance in the mice fed a normal diet were Bacteroidia (65.67% of total abundance), Clostridia (31.27%), and β-Proteobacteria (1.72%). In contrast, the top three classes in order of abundance in the mice fed a high-fat diet were Clostridia (68.05% of total abundance), Bacteroidia (21.66%), and δ-Proteobacteria (7.02%). In the HFD+LGG group, supplementation with low-dose LGG increased the population of Clostridia to 70.86% and decreased the population of Bacteroidetes and δ-Proteobacteria to 14.29% and 0.03%, respectively. Supplementation with high-dose LGG increased the population of Clostridia and Bacteroidetes to 68.28% and 25.03%, respectively, and decreased the population of δ-Proteobacteria to 0.03%. In addition, supplementation with low-dose LGG and high-dose LGG in mice fed a high-fat diet increased Erysipelotrichia to 10.07% and 4.08%, respectively, and increased Bacilli to 3.54% and 1.75%, respectively.

## 4. Discussion

It is well known that a western diet high in fats and sugars and low in fiber leads to the loss of epithelial barrier integrity and increased levels of endotoxin-producing bacteria in the intestinal tracts. This condition causes dysbiosis that further alters epithelial homeostasis and deregulates mucosal inflammation, resulting in metabolic disorders, and finally leads to obesity and type 2 diabetes [20,21,22]. A previous study demonstrated that consumption of a high-fat diet rapidly induced insulin and leptin resistance in rats [23]. Leptin resistance, which is associated with obesity, is a phenomenon like insulin resistance in patients with type 2 diabetes.

Leptin resistance is considered to be attributable to several pathways, including hyperleptinemia, failure of central transport of the hormone, endoplasmic reticulum stress, and receptor signaling impairment. However, the exact mechanisms that sustain leptin resistance are still unclear and need to be clarified [24]. In this study, we demonstrated that the mice fed a high-fat diet had higher body weights and epididymal fat weights than mice fed a normal diet. Moreover, exogenous leptin did not result in significant weight loss in mice fed a high-fat diet. In contrast, exogenous leptin treatment caused a marked and significant body weight reduction in mice fed a normal diet. These results are consistent with those of the previously mentioned studies. Although the body weights and epididymal fat weights of mice fed a high-fat diet were similar to those of mice fed a normal diet, supplementation with high-dose LGG in the high-fat diet restored exogenous leptin responsiveness. According to these results, we suggested that LGG possesses the ability to alleviate leptin resistance caused by a high-fat diet.

Several previous studies have reported that the administration of LGG may affect lipid metabolism in mice with HFD-induced obesity. Kim et al. [15] reported that mice fed a high-fat diet supplemented with LGG for 13 weeks had significantly reduced body weight gain, without any effect on epididymal fat weight. Ji et al. [25] reported that mice fed a high-fat diet supplemented with LGG for 10 weeks had significantly reduced HFD-induced body weight gain and epididymal fat weight gain. The inhibitory effect of LGG on lipid accumulation might be attributed to its stimulating effect on adiponectin secretion and the sequential activation of AMP-activated protein kinase (AMPK) [15]. In addition, LGG reduced the expression of hepatic lipogenic enzymes, including acetyl-CoA carboxylase, fatty acid synthase, and stearoyl-CoA desaturase-1 [26,27]. Elevated expression of hepatic lipogenic enzymes is associated with excess lipid accumulation in the liver, which is accompanied by a range of hepatic histological alterations, varying from microvesicular steatosis to macrovesicular steatosis [27]. We found that the liver specimens from the mice fed the HFD+LGG-HD had fewer hepatic lipid droplets and did not exhibit microvesicular and macrovesicular liver steatosis compared with mice fed a standard high-fat diet. We suggested that the reduction in lipid accumulation in the liver might be attributed to the inhibitory effect of LGG on the expression of hepatic lipogenic enzymes or its stimulating effect on adiponectin secretion and activation of AMPK.

Circulating leptin levels are related to body fat mass. The binding of leptin to its hypothalamic receptor stimulates appetite suppression and leads to the inhibition of fat accumulation and reduction in body weight [3]. However, obesity is associated with high circulating leptin levels occurring concomitantly with leptin resistance at the level of the central nervous system. Previous studies have indicated that chronically elevated leptin levels are associated with gradually increasing leptin resistance [4,28,29]. However, the exact mechanisms of leptin resistance are not clear. One of the proposed mechanisms is that the upregulation of *SOCS3* may result in the development of leptin resistance [4]. In addition, previous studies found that exogenous leptin treatment in mice induced higher levels of SOCS3 in the hypothalamus, and mice with obesity induced by a high-fat diet do not respond to exogenous leptin treatment due to leptin resistance [30,31]. We evaluated the effect of administration of LGG on the leptin resistance in an acute mouse model based on the fact that leptin resistance prevents exogenous leptin treatment from promoting weight loss [4]. We found that leptin responsiveness to exogenous leptin treatment was reduced in mice fed a high-fat diet. However, the administration of LGG at a high dose increased leptin responsiveness to exogenous leptin treatment in mice with obesity induced by a high-fat diet. Moreover, the hypothalamic SOCS3 levels in the HFD+LGG-HD group were significantly increased, indicating that the mice fed an HFD+LGG-HD diet were more sensitive to exogenous leptin. Several previous studies have demonstrated that probiotics induce *SOCS3* gene expression [32,33]. However, recent experimental studies have demonstrated that probiotics may decrease the expression of the *SOCS3* gene [34,35]. These conflicting results could be due to differences in the probiotic strains used in the experiments. Further studies are needed to verify the underlying mechanism responsible for the regulatory effects of probiotics on *SOCS3* gene expression and indicate which probiotics are effective for the alleviation of leptin resistance.

The intestinal mucus layer acts as the primary barrier to protect the epithelial cell surface from the invasion of commensal bacteria or pathogens. A western-style diet can alter the intestinal barrier structure via a reduction of tight junction proteins, which increases the penetrability of the intestinal mucous layer. The result is the translocation of microbial products from the gut into systemic circulation, which can trigger metabolic disease [22]. Quantitative histology is, at present, the best research tool for the assessment of histological change in the intestinal mucosa. The ratio of villus height to crypt depth is reported as a useful criterion to estimate intestinal inflammatory disorders in mouse models [36,37]. In the duodenum and jejunum of healthy wild-type mice, the villi are long, with a ratio of villus height to crypt depth in the order of 3:1 to 5:1, whereas the villi in the ileum are typically shorter. In the small intestinal tracts of mice with intestinal inflammation, the villus height is decreased, and the ratio of villus height to crypt depth is less than 2:1, which indicates moderate villous blunting [37]. We found that the ileum of mice fed a high-fat diet showed moderate villous blunting, with a ratio of villus height to crypt depth of 1.76 ± 0.29. The administration of LGG significantly increased the villus height and ratio of villus height to crypt depth to normal levels, indicating that LGG could alleviate the intestinal inflammatory syndrome caused by a high-fat diet.

In the vertebrate gut, the dominant two bacterial phyla observed are Bacteroidetes (particularly the genera *Bacteroides* and *Prevotella*) and Firmicutes (particularly *Clostridium* and *Lactobacillus*), which constitute 80–90% of the resident bacteria. The two other phyla present but less abundant are Proteobacteria (particularly *Escherichia*) and Actinobacteria (particularly *Bifidobacterium*) [8]. Intake of a high-fat diet may modify the intestinal microbiota, characterized by decreased diversity and by an increased ratio of Firmicutes/Bacteroidetes, although the exact mechanism by which a high-fat diet affects the composition of the intestinal microbiota is unclear [20]. Consistent with previous studies, we found that Firmicutes was more abundant than Bacteroidetes in the intestinal microbiota of mice fed a high-fat diet, with a ratio of Firmicutes/Bacteroidetes of 3.24, which is higher than that of mice fed a normal diet (0.49). Moreover, the microbial proportion of the phyla Proteobacteria was higher in the intestinal microbiota of mice fed a high-fat diet than that of mice fed a normal diet. Supplementation of the high-fat diet with high-dose LGG did not decrease the proportion of Firmicutes but increased the proportion of Bacteroidetes and decreased the proportion of Proteobacteria. Since an increased prevalence of Proteobacteria is a potential diagnostic signature of dysbiosis and risk of disease [38], LGG supplementation could improve the digestive health of the host through its ability to reduce proteobacterial populations in intestinal microflora.

Recently, several studies concluded that the risk of major adverse cardiovascular events increased with the elevation of the plasma trimethylamine N-oxide (TMAO) level [39,40]. Elevation of the plasma TMAO level has also been linked to substantially increased risk of type 2 diabetes and metabolic syndrome. Moreover, TMAO may serve as a marker for hepatic insulin resistance [41]. TMAO is derived from the gut microbial metabolism of dietary choline to trimethylamine (TMA), which is further converted in the liver into TMAO [41]. In this study, we found that LGG supplementation might alleviate leptin resistance caused by an HFD through the improvement of gut microbiota dysbiosis. Future studies will be aimed at identifying the relationship among plasma TMAO level, gut microbiota, and leptin resistance.

## 5. Conclusions

Our results showed that the mice fed a high-fat diet had higher body and epididymal fat weights and greater leptin resistance, including the lack of weight loss and higher hypothalamic *SOCS3* expression levels in response to exogenous leptin treatment, a lower ratio of villus height to crypt depth, and a higher proportion of Proteobacteria and ratio of Firmicutes/Bacteroidetes in the fecal microbiota than mice fed a normal diet. Supplementation of the high-fat diet with a high dose of LGG increased serum leptin concentrations and restored exogenous leptin responsiveness, increased the ratio of villus height to crypt depth, and decreased the proportion of Proteobacteria in fecal microbiota. According to these results, we suggest that LGG supplementation can alleviate leptin resistance caused by HFD through improvement of the digestive health of the host.
